# Flavonoids and miRNA Modulation: A Novel Approach to Managing Cancer, Diabetes, Depression, and Neurological Disorders

**DOI:** 10.1155/bmri/6626244

**Published:** 2026-07-08

**Authors:** Mohadese Mahdie, Sedigheh Momenzadeh, Somayeh Reiisi, Razieh Heidari

**Affiliations:** ^1^ Department of Genetics, Faculty of Basic Sciences, Shahrekord University, Shahrekord, Iran, sku.ac.ir; ^2^ Department of Medical Biotechnology, School of Paramedicine, Bushehr University of Medical Sciences, Bushehr, Iran, bpums.ac.ir; ^3^ Cellular and Molecular Research Center, Basic Health Sciences Institute, Shahrekord University of Medical Sciences, Shahrekord, Iran, skums.ac.ir

**Keywords:** cancer, chronic diseases, depression, diabetes, flavonoids, microRNAs

## Abstract

microRNAs (miRNAs) regulate gene expression post‐transcriptionally and play essential roles in various cellular processes. Disruption of their normal expression contributes to the pathogenesis of numerous diseases, including cancer, diabetes, depression, and neurological disorders. Consequently, restoring or inhibiting miRNA function has become a major focus of research, utilizing advanced technologies. Despite their potential, the clinical application of these technologies remains limited due to challenges related to delivery, specificity, cost, and safety. In contrast, growing evidence suggests that natural compounds—particularly flavonoids, a class of polyphenolic compounds abundant in plant‐based foods such as fruits, vegetables, tea, and whole grains—can beneficially modulate miRNA expression. Studies across various disease models have demonstrated that flavonoids influence disease‐related pathways through miRNA regulation, showing promising results in reducing disease complications, especially in preclinical mouse models. Therefore, diets rich in flavonoids are not only beneficial for disease prevention but also hold potential therapeutic value through the targeted modulation of miRNAs. The effectiveness of flavonoids across a wide spectrum of chronic diseases introduces a novel class of miRNA modulators with significant potential for clinical application. This review comprehensively examines recent studies on the use of various flavonoids in cellular models, animal models, and human tissue samples related to cancer, diabetes, depression, and neurological disorders. It further explores the molecular mechanisms through which flavonoids regulate miRNA expression, including transcriptional control, epigenetic modifications, and modulation of signaling pathways. Additionally, the review discusses the therapeutic opportunities and challenges associated with flavonoid use and proposes potential strategies to overcome these limitations.

## 1. Introduction

As chronic diseases such as diabetes, cancer, and neurological disorders become more prevalent, it is of particular interest to achieve effective treatments. Cancer constitutes a grave health issue globally [1]. Modern lifestyle and diet have led to the prevalence of metabolic diseases such as diabetes and obesity, which have inflicted considerable economic and social costs [2]. Depression is a common and debilitating psychiatric disorder. Although various types of antidepressants exist, novel drugs with greater effectiveness and fewer side effects are needed [3]. As life expectancy increases, neurodegenerative diseases are becoming more widespread. These diseases, such as Parkinson’s, amyotrophic lateral sclerosis (ALS), and Alzheimer’s, primarily impact the elderly [4]. Although different treatments are available for these diseases, the discovery of more effective therapeutic approaches and the improvement of the existing ones continue. Research has recently focused on nutritional interventions and natural compounds to control and manage these diseases considering their fewer side effects, accessibility, low cost, and potential to regulate cellular and molecular pathways especially gene expression. Regarding this, the research process can be accelerated by understanding the pathogenesis of these disorders and their related molecular mechanisms. Recent years have seen significant interest in various molecular pathways, particularly epigenetic regulation through microRNAs (miRNAs). miRNAs are noncoding RNAs responsible for the post‐transcription gene expression through binding to complementary sequences in target mRNAs [5]. The regulatory mechanism of miRNAs occurs mainly via base‐pairing with complementary sequences in the 3 ^′^ untranslated regions of target mRNAs, which leads to translation repression or mRNA degradation. Given the effect of these molecules on basic cellular processes such as proliferation, differentiation, apoptosis, and immune responses [6], alterations in their expression correspond to the onset and progression of different diseases. Changes in the expression of miRNAs have been indicated in cancer [7, 8], diabetes [9], depression [10], and neurodegenerative diseases [11]. Therefore, therapeutic approaches with the aim of miRNA activity modulation are greatly promising for the treatment of different diseases. Different strategies have been considered as tools to target miRNAs, including antisense oligonucleotides, miRNA sponges, and CRISPR/Cas9 genome editing. However, research in this field is becoming more extensive, the clinical implementation of these techniques has been hampered by challenges related to their effective delivery. To address this, naturally derived compounds have received the interest of researchers due to their ability to affect gene regulation. However, relatively limited research has been devoted to investigate the impact of herbal compounds on miRNA expression [12]. A growing body of research in the field of cancer, diabetes, depression, and neurodegenerative diseases shows that flavonoids are natural bioactive compounds with considerable anti‐inflammatory and antiproliferative effects. Encompassing a large group of polyphenolic compounds widely present in fruits, vegetables, and plant‐based beverages, flavonoids show a broad range of biological activities, due to which they are now considered as potential epigenetic modulators with the ability of changing the expression of miRNAs. Given their therapeutic potential observed both in vitro and in vivo models, these compounds are of value in miRNA‐based therapies [13–15]. In this review study, the goal is to provide an extensive overview of current knowledge regarding miRNA expression modulation by flavonoids and to assess their potential in treating cancer, diabetes, depression, and neurodegenerative disorders. Moreover, this review also addresses existing restraints and future approaches for the clinical implementation of flavonoid‐based miRNA therapies.

This article is a narrative review that synthesizes current evidence on flavonoid‐mediated modulation of miRNAs and their relevance in cancer, diabetes, depression, and neurological disorders. The search strategy employed combinations of keywords such as flavonoids, miRNA, miRNA regulation, cancer, diabetes, depression, and neurological disorders. A structured literature search was conducted in major databases covering publications from 2000 to 2025. Studies were selected based on relevance, focusing on peer‐reviewed articles addressing miRNA‐related mechanisms, while nonrelevant sources were excluded.

## 2. Effect of miRNAs Regulation on Health and Diseases

Gene expression regulation by miRNAs occurs through either the degradation of target mRNAs or the inhibition of their translation. Research so far has mainly revealed the interaction of miRNAs with specific sequences located in the 3 ^′^ untranslated region (3 ^′^ UTR) of target mRNAs and the resulting inhibition of translation together with the removal of the poly(A) tail and the 5 ^′^ cap of the mRNA [5]. After transcription of miRNAs by RNA polymerase II as longer primary transcripts (pri‐miRNAs), pri‐miRNAs are first processed by the Drosha–DGCR8 complex in the nucleus to generate pre‐miRNAs, which are then exported to the cytoplasm via exportin‐5. In the cytoplasm, Dicer cleaves pre‐miRNAs into ~22‐nucleotide miRNA duplexes that are subsequently loaded into the RNA‐induced silencing complex (RISC). In this regard, the RISC complex, which is composed of the guide strand of miRNA and the Argonaute (AGO) protein, suppresses gene expression via establishing a bond to complementary regions on the mRNA. In case this binding is completely complementary, mRNA cleavage occurs through AGO [16]. Guide strand selection versus passenger strand incorporation is influenced by the thermodynamic asymmetry of the duplex ends and interactions with AGO proteins. Typically, the strand with lower 5 ^′^ end stability is preferentially selected as the guide strand, while the opposite strand is degraded; however, in some cases, both strands can be functionally active [17]. Several intrinsic and extrinsic factors can contribute to the expression and processing of miRNAs. miRNAs are able to interact with hundreds of mRNAs; thus, they produce complex regulatory networks that affect almost all cellular processes [5]. miRNA regulation is mainly driven by genetic and epigenetic factors such as mutation in miRNA‐coding genes, variation in their regulatory regions, and single nucleotide polymorphisms, which can impact the function and expression of miRNAs. Epigenetic modifications include DNA methylation, post‐translation histone changes, and RNA editing. Interestingly, epi‐miRNAs, a subset of miRNAs, have the ability to affect the function of key epigenetic enzymes, such as DNA methyl transferases, histone deacetylases, and histone methyl transferases, and, as a consequence, modify both protein‐coding and noncoding gene expression and contribute to broad changes in the epigenetic landscape. Emerging evidence suggests that, in certain contexts, miRNAs may localize to the nucleus and interact with complementary sequences within gene promoters, potentially influencing transcription by modulating chromatin architecture or recruiting regulatory protein complexes; however, these noncanonical functions are not yet fully understood and remain an area of ongoing investigation [5, 18]. The normal miRNA gene regulation can be disrupted by genetic or epigenetic changes, such as DNA methylation modifications, histone modifications, miRNA‐encoding regions mutations, or biogenesis machinery changes [19]. Anomalous expression profiles of miRNAs have been consistently discovered in cancers, neurological diseases, cardiovascular disorders, and metabolic conditions. In this regard, certain miRNAs may serve as oncogenes or tumor suppressors [20], or as regulators of insulin signaling [21], neuronal plasticity [22], and inflammatory responses [23]. Through the post‐transcriptional alteration of these processes, miRNAs are able to either lead to further progression of a disease or, on the contrary, serve as protection mechanisms with respect to their expression patterns. Given the involvement of miRNAs in such an extensive range of cellular functions, a promising treatment strategy would be to modulate their expression profiles in affected tissues [24]. In this regard, an emergent research strategy is to target miRNA activity, especially in diseases for which conventional treatments have limitations or severe side effects. Research has shown that certain nutritional and phytochemical factors, such as flavonoids, are able to affect the expression of miRNAs and can lead to novel dietary or plant‐based treatments.

## 3. Flavonoids: Structure, Sources, and Biological Functions

Flavonoids are polyphenolic secondary metabolites with 15 carbon atoms, which are produced by a wide variety of plants and fungi. Flavonoids are nonessential bioactive compounds that are commonly ingested as glycosides and require enzymatic or microbial hydrolysis prior to absorption; they also undergo extensive phase II metabolism, resulting in low systemic bioavailability and predominantly circulating as conjugated metabolites [25].

Nearly all plant families include these natural components that are generally grouped into six main subgroups according to the structure of their C ring, especially the attachment position of the B ring and the oxidation and saturation level [26, 27]. Nearly all plant families include the natural components that are generally grouped into six main subgroups according to the structure of their C ring, especially the attachment position of the B ring and the oxidation and saturation level [27, 28]. Flavonoids have many subgroups, including flavones, isoflavones, flavonols, flavanones, flavanols (catechins), and anthocyanins. In isoflavones, the B ring is attached at the C3 position of the C ring, while in flavones and flavonols, the B ring is located at the C2 position, with the latter having a hydroxyl group at the C3 position. Moreover, flavanones feature a saturated C2–C3 bond, and although flavanols share the same saturation, they possess a hydroxyl group at the C3 position. Anthocyanins, usually associated with pigmentation in plants, are glycosylated derivatives that have an oxygen atom with a positive charge in the C ring [28] (Table 1).

**Table 1:** Flavonoids properties and classification.

Chemical subclass	Chemical structure	Water solubility	Example of compounds	Major food source	Effect	Refs.
Isoflavones	3‐Phenylchromen‐4‐one	Low	Daidzein, Genistein, Formononetin, Alpinumisoflavone	Legumes, soybeans	Antioxidant	[29–32]
Flavanols	2‐Phenyl‐3,4‐dihydro‐2H‐chromen‐3‐ol	Medium	Proanthocyanidins, Epigallocatechin, Epicatechin, Catechin	Tea, apples, grapes, tomatoes, lettuce, onions, cherries	Antioxidant, anti‐inflammatory, free radical scavenging	[33]
Anthocyanidins	Flavylium (2‐phenylchromenylium)	High	Malvidin, Pelargonidin, Delphinidin, Cyanidin, Cyanidin‐3‐glucoside	Grapes, strawberries, berries, cherries	Antioxidant, anti‐inflammatory, anticancer, antibacterial, anti‐angiogenic, cardioprotective	[34, 35]
Flavones	2‐Phenylchromen‐4‐one	Low	Tangeretin, Luteolin, Apigenin, Chrysin, Baicalein, Tricin	Thyme, parsley, tomato skin, red pepper, buckwheat, red wine, fruit skins	Anticancer, anti‐inflammatory, antioxidant, neuroprotective, cardioprotective	[36–41]
Flavanones	2,3‐Dihydro‐2‐phenylchromen‐4‐one	Low	Hesperidin, Naringenin, Naringin, Eriodictyol, Taxifolin	Oranges, lemons, grapefruits, citrus fruits	Anti‐inflammatory, antioxidant	[42, 43]
Flavonols	3‐Hydroxy‐2‐phenylchromen‐4‐one	Low	Quercetin, Kaempferol, Myricetin, Fisetin, Rutin, Morin, Isorhamnetin, Tamarixetin	Tea, grapefruit, berries, cherries, apples, kale, red wine, onions, broccoli	Anticancer, antiviral, antibacterial, cardioprotective, antioxidant	[33, 44–47]

**Figure 1:**
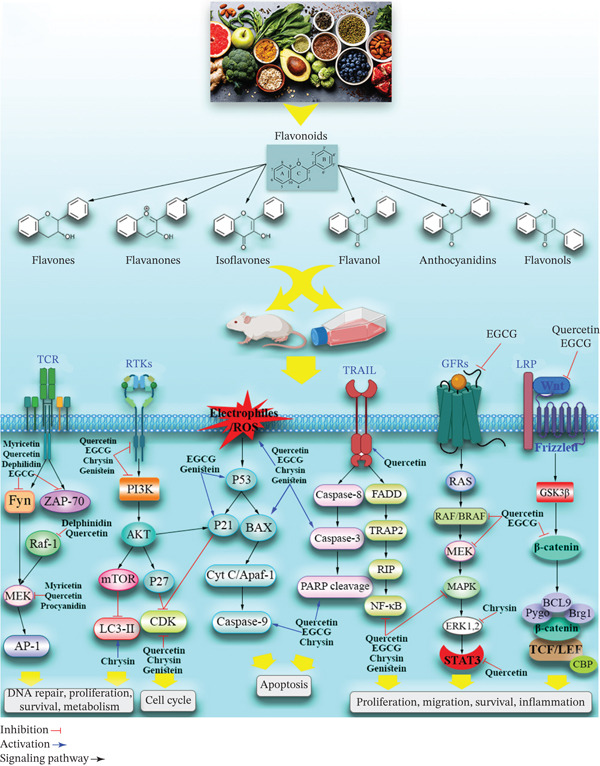
Flavonoids are capable of targeting different signaling pathways in cells contributing to critical biological mechanisms including cell proliferation, cell cycle regulation, differentiation, and apoptosis, both in vitro and in vivo. With respect to cancer treatment, these processes are of particular relevance, given that flavonoids may suppress the growth of tumors and promote the death of cancer cells. Moreover, the anti‐inflammatory and antioxidant aspects of flavonoids may contribute to alleviating symptoms of anxiety and depression along with other chronic diseases such as diabetes.

Given the antioxidant features of flavonoids, they can scavenge reactive oxygen species and thereby protect against oxidative stress and associated diseases [48–50]. Among the subgroups, flavanols, especially catechin and epicatechin, have the most prevalence and are abundant in sources such as tea, grapes, wine, cocoa, and chocolate, which are widely consumed. These flavanols have different chemical structures depending on the source. For example, cocoa has monomers and oligomers of epicatechin (proanthocyanidins), while grapes mainly contain monomeric tannins, and tea mainly features galloylated forms and their oligomers [51]. Flavonoids possess various health benefits, including antioxidant activity [52], cardioprotection [53], capillary resistance improvement [54], body weight regulation [55], allergic response mitigation [56], neuroprotection in age‐related neurodegenerative conditions, and antimicrobial effects [57]. Flavonoids also show anti‐inflammation effects, affect ion transportation, and prevent platelet aggregation. In addition, they have the potential to be used in chemotherapy since they interfere with multiple mechanisms participating in cancer including cell growth proliferation or inhibition by cell cycle restriction, differentiation induction, and apoptosis or a combination of these mechanisms [58, 59]. Besides oncology, flavonoids have also been reported to play a role in mitigating symptoms of anxiety and depression, occurring both as principal psychiatric conditions and as comorbidities in chronic diseases such as cancer, diabetes, and cardiovascular disorders. Their ability to modulate neuroinflammation and oxidative stress drives their beneficial effects [60]. In addition, the neuroprotective impacts of flavonoids in neurodegeneration models have been reported to have low toxicity and mild side effects; this highlights their potential as safe therapeutic agents [4]. Extensive research into the health benefits of flavonoids has resulted in their broad utilization in medical research to develop new therapies based on natural products. Therefore, research in this area has mainly focused on the identification and comprehensive analysis of the mechanisms of action of flavonoids on cellular processes (Figure 1). The literature reports that a potential mechanism by which flavonoids exert their effects is gene expression regulation via the modulation of epigenetic pathways, particularly miRNAs. These compounds are capable of affecting multiple cellular processes and contributing to the regulation of physiological responses by modulating miRNAs expression and function.

## 4. Mechanisms of miRNA Modulation Through Flavonoids

The bioavailability of flavonoids in different tissues is the main driver of their molecular mechanisms of action. According to research, concentrations of flavonoids and their metabolites in vivo are normally smaller than those of other nutrients. They experience comprehensive metabolism in the small and large intestines, and of the flavonoids not absorbed in the upper gastrointestinal tract, a large fraction reach the colon, in which they are broken down into smaller molecules by the intestinal microbiota, which in turn improves their absorption and bioavailability [61–63]. The ingestion of flavonoids mainly occurs in glycoside form, and some aglycones are released in the small intestine [61]. Flavonoids are generally classified as safe compounds; however, their excessive use may lead to side effects such as allergic responses, gastrointestinal issues, anemia, and hepatotoxicity. Based on available evidence, upon consumption, flavonoids may modulate cellular signaling pathways involved in the progression of various diseases, which might partially explain their proposed preventive and therapeutic effects. [64, 65]. Flavonoids have been reported to target miRNAs associated with diseases, which may enable them to modulate key pathological pathways, mitigate oxidative stress and inflammation, and potentially enhance cellular homeostasis. The influence of flavonoids on miRNA expression is modulated through multiple mechanisms, including direct interactions with signaling molecules as well as indirect modulation via epigenetic regulation. In direct interactions, flavonoids can modulate the activity of transcription factors or alter the expression of genes that encode proteins involved in the regulation of miRNA transcription [64]. For instance, genistein has been shown to reduce the expression of genes such as pAK2, LIMK2, ARHGEF6, CFL2, PIK3R3, PLAU, and ANXA2, which are responsible for regulating miR‐23b. Consequently, genistein enhances the expression of miR‐23b, thereby influencing the progression of breast cancer. Indirect interactions, epigenetics, refer to reversible alterations at the cellular and genetic levels that occur without modifications to the underlying DNA sequence. Key mechanisms underpinning these processes encompass DNA modifications, notably methylation; histone modifications, including deacetylation, phosphorylation, and ubiquitination; nucleosome positioning and regulating of noncoding RNAs [66]. Perturbations in the normal functioning of epigenetic pathways can result in dysregulated gene expression, which may contribute to the pathogenesis of various diseases, including cancer. Furthermore, natural compounds, particularly flavonoids, have demonstrated potential in modulating epigenetic abnormalities. One of the most important epigenetic pathways that flavonoids are able to influence and consequently control disease is noncoding RNAs, especially miRNA. For example, quercetin can increase the expression of miR‐146a in breast cancer cells, activate caspase‐3 and Bax, and induce apoptosis through a mitochondria‐dependent pathway, thereby inhibiting cell proliferation. It also reduces cancer cell invasion by inhibiting EGFR [67].

## 5. miRNA Modulation by Flavonoids in Chronic Diseases

### 5.1. Cancer

**Figure 2:**
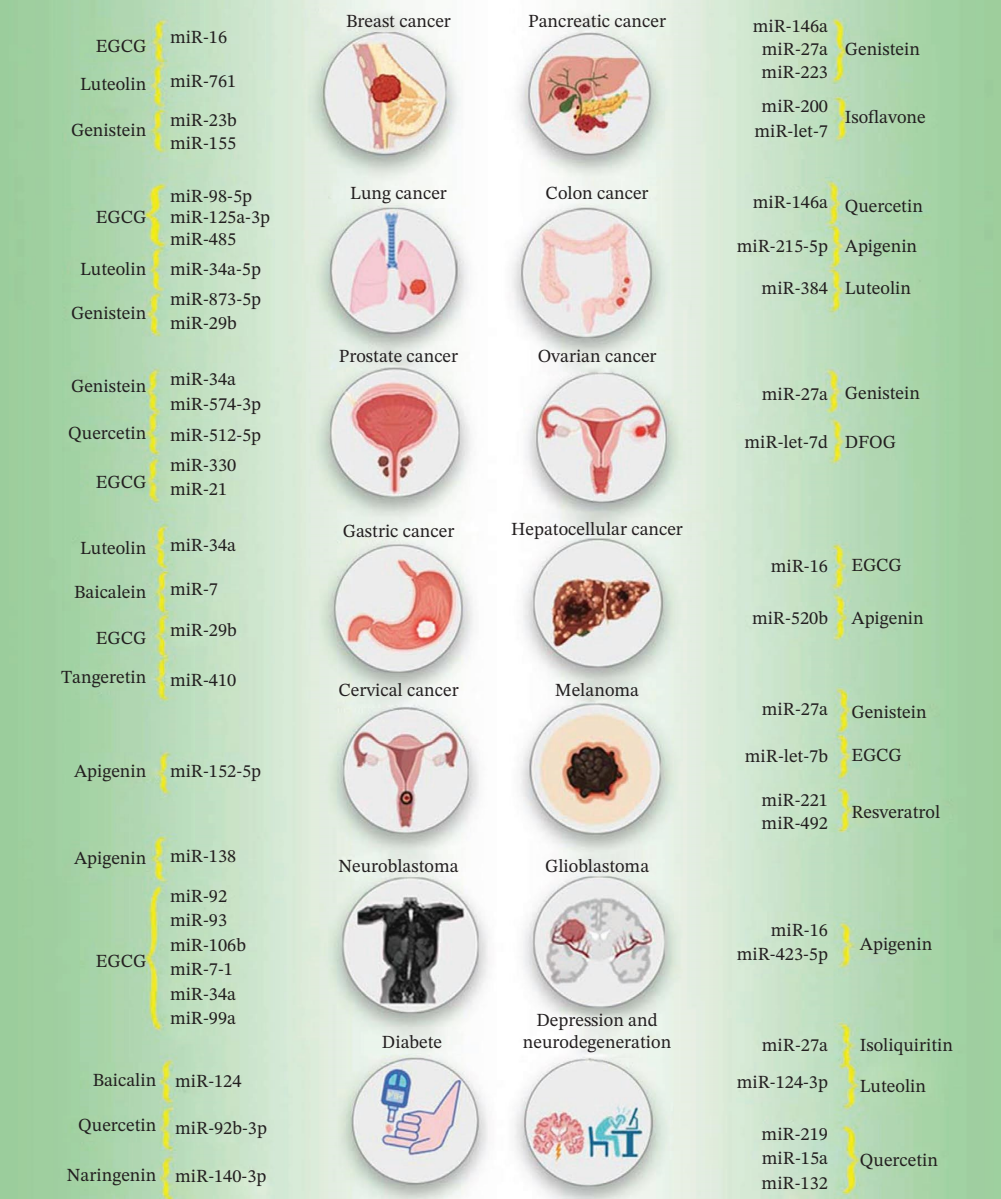
The expression of various microRNAs has been investigated in the presence of different flavonoids across different types of cancer.

Given the potential of flavonoids to interact on various signaling pathways associated with cancer cell proliferation, apoptosis, and metastasis, they may contribute to the development of potentially safer and more effective therapeutic options for cancer. As a principal aspect of cancer research, it is important to find out how flavonoids interact with pathways participating in regulating gene expression and epigenetics, especially via miRNAs (Figure 2). The expression of oncogenic and tumor suppressor miRNAs is modulated by different flavonoids including quercetin, fisetin, apigenin, genistein, and epigallocatechin gallate. For instance, through the PI3K/AKT and NF‐*κ*B pathways, quercetin applies anti‐inflammatory and antiproliferative effects on breast cancer cells by downregulating miR‐27a and miR‐155 [68]. Moreover, Tao et al. also addressed the impacts of quercetin on human breast cancer cells and reported a significant modulating effect of quercetin on the expression of miR‐146a. By upregulation miR‐146a, cell proliferation is inhibited, apoptosis is induced via caspase‐3 activation, and invasion is inhibited by the downregulation of the EGFR expression [67]. Jalalpour et al. reported that utilizing quercetin/fisetin and naringenin in combination enhanced antiproliferative and anti‐migratory outcomes in human breast cancer cell lines of MCF7 and MDA‐MB‐231 through the upregulation of miR‐1275 and downregulation of miR‐27a‐3p [69]. Considering the low expression of miR‐146a in pancreatic cancer cells, its re‐expression can repress the invasion potential of pancreatic cancer cells via downregulating EGFR and the IRAK‐1. Genistein, as a nontoxic miRNA activator, led to the upregulation of miR‐146a and, consequently, suppressed or reduced the invasion and metastasis of pancreatic cancer cells [70]. As reported by Dong‐Hu Zhou et al., EGCG raised the levels of p53 in NSCLC via the downregulation of miR‐98‐5p and miR‐125a‐3p; thus, sensitivity to cisplatin increased [71]. Other miRNAs with increased expressions in lung adenocarcinoma cells due to a quercetin‐rich diet are miR‐146a, miR‐16, and miR‐26 [72]. Claudin‐2, a major membrane protein in the formation of tight junctions, can be regulated by miR‐16. Impairment of cell adhesion is a step in the progression of cancer to metastasis, and claudin‐2 dysregulation has been recorded in various tumor cells [73]. Table 2 provides details of how flavonoids effectively control various signaling pathways by targeting different miRNAs in various cancers. The aforementioned studies examined the potential of flavonoids to modulate the expression of miRNAs and suggested potential new strategies for effective, miRNA‐targeted anticancer therapies. The reported results indicated the nutritional value of flavonoids and their miRNA regulation effects in cancer control. Hence, these compounds may be considered promising candidates for further investigation in cancer prevention and treatment, but only with the following caveats: the current evidence is primarily derived from preclinical models (in vitro and animal studies), human clinical data are sparse; issues such as low bioavailability, metabolic instability, and lack of dose standardization remain unresolved; and potential long‐term safety has not been adequately evaluated. Consequently, translation to clinical practice requires substantial further research.

**Table 2:** The role of flavonoids in modulating miRNA expression across different cancer types.

Cancer	Flavonoid	Analyzed miRNA	MiRNA expression following flavonoid exposure	Some of analyzed genes	Model system (in vitro/animal/human)	Result	Refs.
Breast	Quercetin	miR‐146a	Up	EGFR	MCF‐7 and MDA‐MB‐231 cells/BALB/c nude mice	Suppression of cell proliferation and invasion induction of apoptosis	[67]
	Quercetin, Fisetin and Naringenin	miR‐1275 miR‐27a‐3p	Up Down	Bax and caspase‐3 MMP2, MMP9, Ki‐67, FGF and EGFR	MCF7 and MDA‐MB‐231 cells	Activation of antiproliferative and anti‐migratory activities	[69]
	MQG	miR‐155 and miR‐146a	Down	TP53, MICA/B, ULBP2, CD155, and ICAM1	MCF7, MDA‐MB‐231, Huh7 and Hep‐G2 cells	MQG showed potential to suppress TNBC hallmarks via MALAT‐1, TNF‐*α*/IL‐10 inhibition and TP53 induction, enhancing immune recognition and reducing TME suppression.	[74]
	EGCG	miR‐16	Up	IL6 and TGF*β*	4T1 and RAW264.7 cells. BALB/c nude mice	EGCG upregulated miR‐16, which may transfer to TAMs via exosomes and inhibit TAM infiltration/M2 polarization, suggesting a novel antitumor mechanism in TME.	[75]
	Genistein	miR‐155	Down	P27, CK1*α*, PTEN, and FOXO3	MDA‐MB‐435, Hs578t, and MCF‐7 cells	Inhibition of cell cycling and proliferation through Cyclin D1 suppression, as well as reduction of cytoskeletal remodeling, motility, and metastasis	[76]
	Luteolin	miR‐181a, miR‐139‐5p, miR‐224 and miR‐246	Up	Notch1, HES1, VEGF, MMP2, MMP9 and Cyclin D1	MCF‐7and MDA‐MB ‐231 BALB/c nude mice	Luteolin inhibits breast cancer survival, cell cycle, tube formation, and Notch signaling (protein/mRNA), and modulates miRNAs.	[77]
		miR‐761	Down	E‐cadherin and TINCR	MCF‐10A, MDA‐MB‐468, BT‐549, SUM159PT cells. Serum samples from patients	Imbalance of the TINCR‐miR‐761 module promotes early TNBC metastasis and partially offsets the antitumor activity of luteolin, highlighting a new therapeutic reference for TNBC treatment	[78]
	Hesperidin and Luteolin	miR‐16, miR‐34a miR‐21	Up Up Down	Caspase3, 8, 9 and Bax Bcl2	MCF‐7 cells	Hesperidin and luteolin suppress viability and intrinsic/extrinsic apoptosis via Bcl‐2/Bax, while downregulating miR‐21 and upregulating miR‐16/‐34a (correlated with Bcl‐2)	[79]
	C3G	miR‐138	Down	Sirt1, ZO1 and E‐cadherin	MDA‐MB‐231 and BT‐549 cells	C3G inhibits TNBC migration/invasion via miR‐138 repression and Sirt1 reactivation.	[80]
Pancreatic	Genistein	miR‐27a	Down	Caspase3, and Bax	BxPc‐3 and Panc‐1 cells	Suppress invasion by inhibition of Bcl‐2, MMP2 and VEGF and induced apoptosis	[81]
	Quercetin and Green Tea Catechin and Sulforaphane	miR‐let‐7a	Up	MMP2 and MMP9, ALDH1 and K‐ras	MIA‐PaCa2 and BxPc‐3 cells	Induction of apoptosis and inhibition of self‐renewal potential and migration	[82]
	Quercetin	miR‐let‐7	Up	Ki67, ALDH1, CD24 and CD44	AsPC‐1, CRL‐4023 and PANC‐1 cells Fertilized chick eggs	Quercetin‐induced let‐7c suppresses tumor growth via posttranscriptional activation of Numbl and indirect inhibition of Notch.	[83]
	DIM and isoflavone	miR‐200 let‐7	Up	ZEB1, Slug and Vimentin	MiaPaCa‐2, Panc‐1, Aspc‐1, L3.6pl, Colo357, BxPC‐3 and HPAC cells	DIM and isoflavone function as miRNA regulators that reverse the EMT phenotype in pancreatic cancer	[84]
	Rutin	miR‐877‐3p	Up	Bcl2 and MMP9	PANC‐1, SW1990 and MIA PaCa‐2 cells	Rutin upregulates miR‐877‐3p, which represses Bcl‐2 transcription and induces apoptosis in pancreatic cancer cells.	[85]
	Apigenin	miR‐155	Down	SHIP1 and CD40	MiaPaCa‐2, Panc02 and UN‐KC‐6141 cells Female C57BL/6 mice and SHIPHET mice	Modulating miR‐155 to increase SHIP‐1 expression may enhance antitumor immunity and improve immunotherapy responses in pancreatic cancer.	[86]
	Baicalein	miR‐139‐3p miR‐196b‐5p	Up Down	NOB1 ING5	Panc‐1 cells Balb/c nude mice	Inhibition of cell proliferation, motility and invasion and induction of cell cycle arrest	[87]
Lung	EGCG	miR‐210	Up	—	CL13, H1299, H460 and A549 cells	EGCG elevates miR‐210 via HIF‐1*α* stabilization in lung cancer cells, and this contributes to cancer suppression.	[88]
			miR‐98‐5p and miR‐125a‐3p	Down	P53, RAP2B, MEAF6, PPAPDC2, and SEP11	A549, NCI‐H460, and LTEP‐*α*‐2 cells BALB/c nude mice	Induction apoptosis and increase efficacy of cisplatin
			miR‐485	Up	CD133, CD44, Sox2, Nanog and Oct4	A549 cells BALB/c nude mice	Stemness features and cancer stem cell population were suppressed by EGCG‐modulated miR‐485/CD44.
	Quercetin	miR‐16	Up	Claudin2	A549 cells	Quercetin decreased claudin‐2 in A549 cells. Claudin‐2 and miR‐16 are potential therapeutic targets for adenocarcinomas	[90]
		miR‐16‐5p	Up	Bcl2, Chk1 and WEE1	GLC‐82 cells and HTB‐182	Suppression of proliferation and induction of apoptosis by activation of P53and Bax	[91]
	Luteolin	miR‐34a‐5p	Up	MDM4 and Bcl‐2	A549 and H460 cells BALB/c nude mice	Induces apoptosis by activation of Caspase‐3, Caspase‐9, p21, Bax, and p53	[92]
		miR‐133a‐3p	Up	VEGF, MMP2, MMP9, PI3K, Akt and MAPK	Tissue specimens from patients with NSCLC	Luteolin suppresses NSCLC vascular endothelial cell functions by upregulating miR‐133a‐3p, which downregulates PURB and inactivates MAPK and PI3K/Akt pathways.	[93]
		miR‐106a‐5p	Up	TWIST1 and MMP2	A549 cells	Luteolin inhibits A549 cell migration by modulating the miRNA landscape	[94]
	Genistein	miRNA‐29b	Up	pAKT, p‐PI3K, DNMT3B and MCL 1	A549 cells	Suppression of proliferation	[95]
		miR‐873‐5p	Down	Bax	H292 and A549 cells BALB/c nude mice Tissue specimens from patients with NSCLC	Inhibition of circ_0031250/miR‐873‐5p/FOXM1 axis	[96]
	Apigenin	miR‐34a‐5p	Up	SNAI1	A549 cells	Apigenin might induce apoptosis in A549 cells by upregulating miR‐34a‐5p, leading to downregulation of SNAI1	[97]
	Orientin	miR‐26b and miR‐146a	Up	COX2, PGE2, iNOS and BCL2	MDA‐MB‐231, A549, HCT‐116 and NIH‐3 T3	Induction of apoptosis via CYP‐1A1 expression	[98]
	Acacetin	miR‐34a	Up	PD‐L1, Cyclin B1, Cyclin D, and BCL2	A549 and H460 cells. BALB/c thymic nude mice	Acacetin inhibits proliferation and induces apoptosis in NSCLC cells through the regulation of miR‐34a.	[99]
Colon	Flavonol‐rich fractions of yaupon holly leaves	miR‐146a	Up	NF‐*κ*B, TLR4, IRAK1, TRAF6, IL8, MCP1, PGE2, COX2, AhR, PXR, CYP1A1,CYP1B1	HT‐29 and CCD‐18Co cells	Suppression of NF‐*κ*B activation and activation of GPx, catalase and GST	[100]
	Resveratrol and Quercetin	miR‐27a	Down	Caspase‐3, PARP and ZBTB10	HT‐29 cells	The Sp‐dependent antiapoptotic survival gene survivin was significantly reduced.	[101]
	Luteolin	miR‐384	Up	PTN, MMP2, MMP3, MM9 and MMP16	HT‐29, SW480, SW620, LoVo cells BALB/c thymic nude mice Tissue specimens from patients with CRC	Downregulation of metastasis was also observed.	[102]
	Genistein	miR‐95	Down	SGK1, Bcl‐2	SW620 cells Serum samples from patients with CRC	Genistein may downregulate miR‐95, SGK1, and Erk1	[103]
	Apigenin	miRNA‐215‐5p	Up	Bcl‐2, E2F1 and E2F3	HCT116 and HEK‐293 T cells	Inhibition of miRNA‐215‐5p significantly reduced apoptosis and G0/G1 phase arrest	[104]
Prostate	Genistein	miR‐1296b	Down	CDT1, CDC7, and CDK2	LNCaP and PC3 cells tissue specimens from patients with PC	Activation of minichromosome maintenance gene family by down regulation of MCM2, MCM7, CDC7 and CDT1, the downregulation of oncogenes by miR may contribute to novel therapeutic approaches	[105]
		miR‐34a	Up	HOTAIR	RWPE‐1, LNCaP, PC3 and DU145 cells BALB/c thymic nude mice	Genistein might inhibit prostate cancer cell growth by downregulating HOTAIR, a target of tumor suppressor miR‐34a	[106]
		miR‐151	Down	CASZ1, IL1RAPL1 ARHGDIA, and SOX17	RWPE‐1, LNCaP, PC‐3 and DU145 cells/Tissue specimens from patients	Inhibition of the metastasis and cell progression	[107]
		miR‐574‐3p	Up	EGFR	RWPE‐1, PC3 and DU145 cells/BALB/c thymic nude mice/Tissue specimens from patients	Downregulation of angiogenesis and activation of apoptosis	[108]
		miR‐1260b	Up	sFRP1 and Smad4	DU‐145, RWPE‐1 and PC‐3/Tissue specimens from patients	Genistein downregulates miR‐1260b to target sFRP1 and Smad4, and also modulates them via epigenetics in prostate cancer cells	[109]
	EGCG	miR‐330 miR‐21	Up Down	Androgen receptor	CV1, C4‐2, LNCaP and 22R*ν*1 cells/Female BALB/c thymic nude mice	Inhibition of androgen receptor and the proliferation rate	[110]
	Luteolin	miR‐301	Down	DEDD2	PC3 and LNCaP cells/Tissue specimens from patients	Inhibition of proliferation and induction of apoptosis	[111]
	Quercetin	miR‐512‐5p	Down	XRCC5 and ARv7	C4‐2, 22Rv1, VCaP and 293 T cells/BALB/c thymic nude mice	Quercetin may target the ARv7‐mediated circNHS/miR‐512‐5p/XRCC5 signaling to improve radiosensitivity and suppress prostate cancer progression.	[112]
Ovarian	Genistein	miR‐27a	Down	Sprouty2	SKOV3 cells	Genistein blocks ovarian cancer growth and metastasis by inactivating oncogenic miR‐27a.	[113]
	Quercetin	miR‐145	Up	—	SKOV‐3 and A2780 cells	Induction of apoptosis	[114]
Gastric	Luteolin	miR‐34a	Up	Bcl‐2	BGC‐823 and SGC‐7901 cell. Tissue specimens from patients	Inhibition of cell proliferation and induction of apoptosis	[115]
	Tangeretin	mir‐410	Up	Snail1, Twist1, Notch‐1, Jagged1/2, Hey‐1 and Hes‐1	GES‐1, MGC80‐3, AGS, SGC7901 and MKN45 cells BALB/c thymic nude mice	Tangeretin almost completely inhibits radiation‐induced lung metastasis	[116]
	Chrysin‐PLGA‐PEG NPs	miR‐18a, miR‐21 miR‐221	Down	—	AGS cells	Inhibition of cell proliferation	[117]
		miR‐22, miR‐34a miR‐126	Up	—	AGS cells	Inhibition of cell migration, invasion and chemoresistance	[118]
		miR‐9, Let‐7a	Up	—	AGS cells	Inhibition of growth cells	[119]
	Epigallocatechin‐3‐gallate	miR‐29b	Up	KDM2A	AGS and SGC7901 cells	EGCG upregulates miR‐29b by suppressing LINC00511, leading to apoptosis through the LINC00511/miR‐29b/KDM2A axis.	[120]
	Formononetin	miR‐542‐5p	Down	—	BGC‐823, MNK‐45, SGC‐7901, MGC‐803 cells Male BALB/c thymic nude mice	Suppression of the migration, invasion, and colony formation	[121]
	Baicalein	mir‐7	Up	Focal adhesion kinase	HGC‐27, SGC‐7901, MGC‐803 and BGC‐823 cells	Inhibition of proliferation, metastasis and angiogenesis by mediating of miR‐7/FAK/AKT	[122]
Hepatocellular carcinoma	EGCG	miR‐16	Up	Bcl‐2	HepG2 cells	miR‐16 mediates EGCG’s apoptotic effect and highlights the role of miRNAs in regulating EGCG’s biological activity	[123]
	Apigenin	miR‐101	Down		BEL‐7402 and BEL‐7402/ADM cells	Sensitizing cancer cells to DOX through Suppression of miR‐101/Nrf2 axis	[124]
		miR‐520b	Up	ATG7	BEL‐7402/ADM cells/Patients tissue specimens	Apigenin sensitizes BEL‐7402/ADM cells to doxorubicin by regulating miR‐520b/ATG7‐related autophagy	[125]
Cervical carcinoma	Apigenin	miR‐152‐5p	Down	E‐cadherin	HOSEpiC, C33A, Hela, SiHa, and CaSki CC Patients tissue specimens	Suppression of proliferation, invasion, and EMT by regulation of miR‐152/BRD4	[126]
Melanoma	Genistein	miR‐27a	Down	ZBTB10	C918 cells BALB/c thymic nude mice	Inhibition of cell proliferation	[127]
	EGCG	miR‐let‐7b	Up	HMGA2	Mewo, A375 and B16‐F10 cells	EGCG‐induced upregulation of let‐7b downregulated HMGA2, a tumor progression‐related target gene	[128]
	Resveratrol	mir‐221	Down	TFG	SbCl2, HaCaT, A375, MV3, WM35, SK‐MEL‐3 and SK‐MEL‐5 cells/SCID mice	Downregulation of NF‐*κ*B/miR‐221 and activation of TFG reduce the size of tumor	[129]
		miR‐492	Up	CD147 and Bcl‐2	A375 and SK‐MEL‐28 cells	Regulation of the miR‐492/CD147 pathway mediates resveratrol‐induced apoptosis in malignant melanoma cells	[130]
	Alpinumisoflavone	miR‐124	Up	SPHK1 and COX‐2	A375, SKMEL‐1 and B16‐F10 cells/BALB/c thymic nude mice	Downregulating COX‐2 via the miR‐124/SPHK1 axis reduces metastasis in melanoma	[131]
	Morin	miR‐216a	Up	Wnt3A	MV3 and M14 cells/SCID mice	Suppression of self‐renewal and cell proliferation via inhibiting miR‐216a	[132]
	Licochalcone	miR‐142‐3p	Up	MITF, Rheb	A375, B16‐F10 and HaCaT cells	Activation of autophagy in melanoma cells via miR‐142‐3p/Rheb/mTOR axis	[133]
	Isoliquiritigenin	miR‐301b	Down	C‐PARP, Bax, and caspase‐3	A375 and A2058 cells/BALB/c thymic nude mice	Suppression of cancer cells by targeting miR‐301b/LRIG1 axis	[134]
		miR‐27a	Down	Bax, E‐cadherin, p53 and c‐PARP	A375, A2058, and B16‐F10 cells/BALB/c thymic nude mice	Inhibition of EMT, proliferation metastasis and colony formation and induction of apoptosis	[135]
	Apigenin	miR‐512‐3p	Down	p27 Kip1	WM1361B, WM983A, WM1341D, SK‐MEL‐3, SH‐4, and SK‐MEL‐24 cells/BALB/c thymic nude mice	Suppression of the growth of tumor and cell proliferation	[136]
Neuroblastomas	HPR and EGCG	miR‐92, miR‐93,miR‐106b miR‐7‐1, miR‐34a and miR‐99a	Down Down Down Up Up Up	Bax, caspase‐8, caspase‐3, ICAD, tBid, calpain,SBDP Bcl‐2, N‐Myc, Notch‐1, Id2, and PCNA	SK‐N‐BE2 and IMR‐32 cells	Suppression of the growth of tumor and cell proliferation	[137]
	Resveratrol	miR‐137	Up	EZH2, H3K27me3	Neuro‐2a (N‐2a) cells	Resveratrol ‐induced apoptosis and tumor suppression occur through an epigenetic mechanism that involves repression of EZH2 by miR‐137	[138]
Glioblastoma	Apigenin	miR‐16	Up	Bcl‐2, NF‐*κ*B and MMP‐9	U87 cells	Suppression of proliferation	[139]
	Amentoflavone	miR‐124‐3p	Up	Bcl‐2, DNMT1	U87, LV229, U251, LN18 and U373 Patients tissue specimens	Activation of the apoptosis and inhibition of glycolysis in the glioma cells	[140]
	Tricin	miR‐7	Up	FAK, MMP3 and MMP9	C6 cells	Inhibition of the invasion by activation of E‐cadherin and proliferation	[141]
	Xanthohumol	miR‐4725‐3p	Up	Stim 1	U87‐MG cells	Reduction of cell invasion through inhibiting Stim 1 expression and regulate kinas/c‐Fos pathway	[142]
	Cyanidin‐3‐O‐glucoside	miR‐214‐5p	Up	CTNNB1	LN‐18/TR cells/BALB/c thymic nude mice	Upregulation of miR‐214‐5p reverses chemotherapy resistance in cells by inhibiting the *β*‐catenin/MGMT pathway	[143]
	PSPD3R	miR‐20b‐5p	Down	Atg7 and LC3‐II	U251, BV2, MLE‐12, BV2, MHS and A172 cells/BALB/c thymic nude mice	Inhibition of proliferation by inducing miR‐20b‐5p/Atg7‐dependent autophagy	[144]
	Rutin	miR‐125b	Down	—	GL15 and C20 cells	Suppression of tumor progression and inflammatory responses by inhibition of IL‐6, TNF, and STAT3	[145]

Abbreviations: 4‐HPR (N‐(4‐hydroxyphenyl) retinamide), C3G (cyanidin‐3‐glucoside), DFOG (7‐difluoromethoxyl‐5,4 ^′^‐di‐n‐octylgenistein), DIM (3,3 ^′^‐diindolylmethane), EGCG (epigallocatechin‐3‐gallate), MQG (methoxylated quercetin glycoside), PSPD3R (purple sweet potato delphinidin‐3‐rutin), SFI (skullcapflavone I), TAM (tumor‐associated macrophages).

### 5.2. Diabetes

As a metabolic syndrome, diabetes mellitus, not only causes complications for the patient in the present but can also lead to long‐term vascular problems such as kidney damage (nephropathy), retinal blood vasculature damage (retinopathy), and nerve damage (neuropathy) [2]. These complications arise from complex metabolic alterations and disruptions in different organs and contribute to the overall mortality and morbidity corresponding to the disease. Growing research points to the major role played by miRNAs in the development and progression of diabetic complications. Accordingly, the focus of recent research has increasingly been on the ability of flavonoids to treat complications related to diabetes. It has been reported that flavonoids have modulatory effect on miRNA expression and, thus, may mitigate the adverse impacts of diabetes on critical organs and tissues. Moreover, since flavonoids are considered potentially safe and effective candidates to complement conventional treatments, with fewer side effects, they may represent promising candidates for further investigation as complementary therapies for diabetes [146]. Flavonoids have been revealed as natural miRNA regulators in diabetic tissues. In this regard, it was reported that Fla‐CN, a kaempferol derivative, increased the expression of miR‐27 and pAMPK in liver and adipose tissues, thereby suppressing adipogenic genes and improving glucose metabolism [147]. Baicalin works by upregulating miR‐124, and since miR‐124 targets TLR4, NF‐*κ*B‐mediated renal fibrosis is reduced in diabetic nephropathy [148]. Naringenin led to the improvement of gestational diabetes via the downregulation of miR‐140‐3p, which in turn restored insulin receptor signaling by upregulating IR‐*α* and IGF1R [149]. It was also reported that by downregulating miR‐34a, dihydromyricetin (DHM) led to the suppression of high glucose levels in both cardiomyocytes and the heart tissue of diabetic mice. This miR‐34a downregulation led to the improvement of the defective autophagy and, in turn, the amelioration of diabetic cardiomyopathy (DCM) [150]. Wenjun Li et al. noted that by suppressing the expression of miR‐34a, DHM is able to protect retinal pigment epithelial cells against apoptosis and oxidative stress induced by high glucose. By lowering the levels of ROS, increasing the activity of antioxidant enzymes (SOD and CAT), and elevating the concentration of GSH, DHM ultimately enhanced cell survival under high glucose conditions [151]. Other studies have shown that isorhamnetin enhances insulin sensitivity in skeletal and adipose tissues by increasing the expression of miR‐1 and miR‐3163 and at the same time reducing mTOR and IGF1‐R [152]. In addition, it was found that apigenin alleviates inflammation and fibrosis in diabetic nephropathy through the miR‐423‐5p/USF2 axis, implicating its therapeutic capability in renal conditions [153]. The available evidence indicates that flavonoids may influence miRNA‐regulated signaling networks, which could be associated with attenuation of diabetes‐related complications such as tissue injury and insulin resistance. However, these conclusions are primarily based on preclinical models, and their translational relevance remains limited. In particular, issues such as poor systemic bioavailability, extensive metabolic transformation into conjugated derivatives, variability in flavonoid preparations, and the scarcity of well‐controlled human studies investigating miRNA‐mediated effects collectively constrain the interpretation of the current findings and warrant further investigation. Table 3 summarizes research in this area.

**Table 3:** The role of flavonoids in regulating miRNA expression in diabetes research.

Flavonoid	Analyzed miRNA	MiRNA expression Following flavonoid exposure	Some of analyzed genes	Model system (in vitro/animal/human)	Result	Refs.
Fla‐CN	miR‐27	Up	SREBP‐1c and C/EBP*α*	Male C57BL/6 mice	Induction of anti‐obesity and antidiabetic effects through activation of p‐AMPK and S6K1	[147]
Baicalin	miR‐124	Up	TLR4, COLIV, FN, p‐I*κ*B*α*, p‐p65	HK‐2 cells/Male C57BL/6 mice	Inhibition of renal fibrosis through microRNA‐124/TLR4/NF‐*κ*B axis	[148]
Naringenin	miR‐140‐3p	Down	IR‐*α* and IGF1R	HTR‐8/SVneo, HEK293 and HUVEC cells/Tissue specimens from patients with GDM	Protection of endothelial and trophoblasts cells from the harmful high glucose environment	[149]
DHM	miR‐34a	Down	Caspase‐3, Bax, SQSTM1/p62	Male Wistar rats	Activation of autophagy via miR‐34a suppression and consequently inhibition of MAP1LC3B, Beclin‐1, Bcl‐2 and P53	[150]
	miR‐34a	Down	Superoxide dismutase (SOD), catalase (CAT), Bax, caspase‐3, caspase‐9	ARPE‐19 cells	Induction of apoptosis and glucose‐induced oxidative stress by inhibiting miR‐34a	[151]
Isorhamnetin	miR‐3163 and miR‐1	Up	m‐TOR, IGF1‐R, LncRNA‐RP11‐773H22.4	Male Wistar rats	Isorhamnetin reduced mTOR expression at both molecular and protein levels, supporting its role in ameliorating insulin resistance in type 2 diabetes	[152]
Apigenin	miR‐423‐5p	Down	USF2, E‐cadherin	Male Wistar rats	Suppression of EMT, renal fibrosis and inflammation by regulating miR‐423‐5p‐USF2 axis and downregulating IL‐6, IFN‐*γ*, TNF‐*α*, IV‐C, FN, Col I, vimentin and *α*‐SMA	[153]
Anthocyanins	miR‐182	Down	OGG1	ARPE‐19 cells/Male Sprague Dawley (SD) rats	Inhibition of the progression of diabetic retinopathy via ROS/ERS/miR‐182/OGG1 axis	[154]
EGCG and Quercetin	miR‐16‐5p	Down	Apaf‐1, caspase‐9 and caspase‐3	NIT‐1 cells	Induction of antidiabetes effects	[155]
Quercetin	miR‐92b‐3p	Up	EGR1	T2DM mice	Enhancing miR‐92b‐3p or reducing EGR1 improves IR and pancreatic histopathology in T2DM mice	[156]
Luteolin	miR‐124	Up	C/EBPA	Male Wistar rats	Suppression of neuroinflammation associated with diabetes via moderating miR‐124/C/EBPA axis	[157]

Abbreviations: DIM (3,3 ^′^‐diindolylmethane), Fla‐CN (3‐O‐[(E)‐4‐(4‐cyanophenyl)‐2‐oxobut‐3‐en‐1‐yl]kaempferol).

### 5.3. Depression

According to the World Health Organization, depression is a widespread mental disorder and a major public health issue with symptoms including feeling of guilt, low self‐esteem, agitation, loss of interest, lethargy, decreased appetite, and difficulties with decision‐making and concentration [158]. Dietary flavonoids, as natural compounds with antioxidant and anti‐inflammatory features, may have a significant contribution to the prevention and treatment of depression by reducing oxidative stress and neuroinflammation, both linked to depressive disorders.

Min Li et al. investigated the potential effect of hesperidin on the improvement of depression‐like behaviors induced by lipopolysaccharide in mice and saw a rise in pro‐inflammatory cytokines IL‐1*β*, IL‐6, and TNF‐*α*, along with a drop in the expression of miR‐132 after administrating LPS. It seems that the antidepressant impacts of hesperidin arise from the drop in these pro‐inflammatory cytokines through the miR‐132 pathway, because of a rise in this miRNA in the brain [159]. Yuanjie Li et al. addressed the potential impact of isoliquiritin, a flavonoid, on depression and found that pretreatment with isoliquiritin led to the protection of primary microglial cells against NLRP3 inflammasome activation induced by LPS and ATP. This protective impact was indicated by a drop in proteins associated with inflammation such as NLRP3, p‐NF‐*κ*B, cleaved Caspase‐1, IL‐1*β*, and GSDMD‐N. Moreover, administering isoliquiritin elevated the miR‐27a expression and lowered the mRNA and protein levels of spleen tyrosine kinase [160]. Yunfeng Ren et al. reported that by modulating the miR‐22‐3p/SIRT1 signaling pathway, phenolic compounds derived from apples are able to mitigate cognitive impairments, depression‐like behaviors, and anxiety‐like behaviors. In this work, to develop depression‐like behaviors and mental disorders, lead acetate (Pb(Ac)_2_) was administered to mice, which led to higher oxidative damage to cells, more pro‐apoptotic proteins, increased levels of pro‐inflammatory cytokines IL‐1*β*, IL‐6, and TNF‐*α*, and lower Bcl‐2 expression in the brain. Treatment with apple‐extracted phenolic compounds lowered Pb(Ac)_2_‐induced alterations through controlling oxidative stress, neuroinflammation, and apoptosis via the miR‐22‐3p/SIRT1 pathway [161]. Qing Zhou et al. addressed the effect of luteolin on breast cancer‐related depression and reported that this this treatment prevented neuronal pyroptosis in the hippocampus by regulating the miR‐124‐3p/TNF‐*α*/TRAF6 axis. Treatment with luteolin not only upregulated miR‐124‐3p and downregulated TNF‐*α* and TRAF6 but also lowered NF‐*κ*B phosphorylation and I*κ*B [162]. Current evidence suggests that flavonoids may modulate the expression of certain mood‐related miRNAs and genes, potentially contributing to alterations in neural plasticity and the stress response. However, these observations are largely derived from preclinical studies and limited human data, and do not establish causality. Importantly, many in vitro findings rely on supraphysiological concentrations of unmetabolized flavonoids, whereas in vivo exposure is characterized by low circulating levels of conjugated metabolites, which may differ in biological activity. As such, while flavonoids may have potential as supportive nutritional factors, their efficacy and underlying mechanisms in depression remain to be fully elucidated and require further rigorous, physiologically relevant investigation [163, 164].

### 5.4. Neurodegenerative Disorders

According to the definition, neurodegenerative disorders, which threaten the aging population, are a group of diseases that gradually lead to neuronal disintegration and death [165]. The primary causes of these diseases, including Alzheimer’s, Parkinson’s, and ALS, are neuroinflammation and oxidative stress. To address this, flavonoids can be employed as therapeutic agents given their strong anti‐inflammatory, neuroprotective, and antioxidant properties, as well as their modulatory effects on miRNA and gene expression [4]. Yang, Shengyi et al. supplemented aged mice with cognitive impairment with quercetin and observed increased expression of miR‐219, miR‐15a, and miR‐132 and modulated tau phosphorylation [166]. In another study, Ebrahimian et al. investigated the improving effect of quercetin‐conjugated superparamagnetic iron oxide nanoparticles (QCSPIONs) on cognitive impairments originating from diabetes. It was observed that administering quercetin together with QCSPIONs to diabetic rats reduced inflammation‐related miR‐146a and miR‐9 genes and ameliorated cognitive impairment [167]. Kamran Far et al. examined the effect of *Allium jesdianum*, which contains flavonoids and polyphenolic compounds, on Alzheimer’s disease. According to the results, administering *A. jesdianum* extract led to a significant improvement in cognitive dysfunction and mitochondrial toxicity in male Wistar rats through downregulation of the Bax gene and upregulation of miR‐330, miR‐132, and Bcl‐2; thus, *A. jesdianum* shows promise for improving Alzheimer’s disease and reducing neuronal apoptosis [168]. Therefore, flavonoids may exert neuroprotective effects. One potential mechanism is the modulation of miRNA expression. However, this does not mean that all protective effects are mediated exclusively via the miRNAs. By restoring normal miRNA expression, flavonoids may mitigate neuroinflammation, restrain oxidative damage, and boost neuronal protection and regeneration, making them promising options for alleviating neurodegenerative diseases. Nevertheless, most of these findings are derived from animal studies, and further clinical trials are needed.

## 6. Flavonoids and miRNAs: Opportunities and Challenges

Researchers have intensified their quest for highly effective therapeutic agents with minimal side effects in recent years, which has led to the attraction of naturally derived compounds. Among these compounds, flavonoids are a group of polyphenolic substances that have become promising options given their anti‐inflammatory, antioxidant, anticancer, and gene‐regulatory features. In addition to the ability to affect multiple essential cellular pathways, flavonoids are also uniquely capable of modulating miRNAs as crucial factors in cellular homeostasis and gene expression.

Although flavonoids present positive bioactivities, their clinical administration accompanies multiple issues, including poor solubility in water, low bioavailability, fast metabolism and clearance, and weak tissue‐targeting ability, especially across the blood–brain barrier [169–171]. Considering that each person responds differently to flavonoid administration, the accurate assessment of their health outcomes is challenging. The observed discrepancies are mainly associated with genetic factors, gut microbiota variations, and different metabolic capacities for processing flavonoids. Although research indicates that the relationship between flavonoids and the gut microbiome is bidirectional, this relationship has not yet been explored thoroughly in clinical trials. Despite the potential significant role of genetics in variations among individuals in the absorption and metabolism of flavonoids, the role of specific genes has received little attention. Addressing these research gaps is of particular interest for developing more effective dietary recommendations for different demographic subgroups. The presence of comprehensive preclinical data in this area indicates that conducting large‐scale, randomized, and controlled clinical trials is urgently needed to assess the efficacy, safety, and pharmacokinetics of flavonoids in human populations. Such research efforts would enable practitioners to incorporate flavonoid‐based therapeutic strategies into conventional medicine, either as separate therapies or in combination with current ones [61, 172]. A serious challenge to the therapeutic application of flavonoids is their bioavailability, i.e., the fraction of active substance entering systemic circulation after ingestion, which is affected by factors including gut microbiota composition, chemical structure, and dietary interactions. The absorption of these compounds is considerably affected by structural aspects such as the position and number of hydroxyl groups, saturation of the C‐ring, and the glycosylated or aglycone form of the compound; aglycones normally show greater bioavailability. Moreover, by converting flavonoids into metabolites, either active or inactive, gut microbiota also has a critical role, and differences in microbial composition among individuals can induce variable responses. Furthermore, absorption is improved when consumed in conjunction with dietary fats, whereas it may be reduced due to competition with other polyphenols [64, 65]. Following ingestion, flavonoids undergo extensive phase I and phase II metabolism in the small intestine and liver, yielding conjugated metabolites including glucuronidated, sulfated, and methylated derivatives [25, 173, 174]. These circulating metabolites differ substantially from the parent aglycone compounds typically tested in experimental settings. Importantly, conjugated metabolites often exhibit distinct biological activities, different molecular targets, and significantly, lower potency compared to their parent forms. In some cases, metabolites may be biologically inactive or exert effects through entirely different mechanisms. Consequently, in vitro studies that expose cultured cells to unconjugated aglycones at supraphysiological concentrations may not accurately reflect the in vivo situation following dietary flavonoid consumption [175–178]. A further layer of complexity arises from the bidirectional interaction between flavonoids and the gut microbiota. Gut bacteria metabolize flavonoids into a diverse array of low‐molecular‐weight phenolic compounds that may contribute significantly to their biological effects. For instance, protocatechuic acid (PCA), a major microbial metabolite of cyanidin‐3‐glucoside, has been shown to exert antiatherogenic effects partially through modulation of the miRNA‐10b–ABCA1/ABCG1 cholesterol efflux pathway. However, despite such emerging evidence, the majority of gut microbiota–derived flavonoid metabolites remain insufficiently characterized, particularly with respect to their roles in miRNA regulation. This gap highlights the need for future studies to systematically investigate physiologically relevant metabolite profiles and their mechanistic involvement in gene regulatory networks [179].

Another challenge concerning the application of flavonoids is that the concentrations used in research often differ from those consumed in the diet. Typical postprandial plasma concentrations of flavonoid metabolites in humans are generally in the low nanomolar to sub‐micromolar range (generally less than 1 *μ*M), whereas a substantial portion of in vitro literature reports bioactivity at micromolar concentrations (10–100 *μ*M or higher) of non‐metabolized parent aglycones. This concentration gap represents a fundamental limitation in directly translating preclinical findings to human physiology [180–188].

A critical distinction must be made between flavonoids consumed as part of a regular diet versus those administered as high‐dose dietary supplements. Dietary flavonoids, at intake levels typical of plant‐rich diets (approximately tens to several hundred mg/day), have been associated with favorable health outcomes in epidemiological studies without significant safety concerns. In contrast, high‐dose supplementation (typically several hundred milligrams up to 1 g/day for individual flavonoids in human supplementation studies) has been reported to achieve systemic concentrations sufficient to modulate biological activity [189–194]. These concentrations may achieve systemic levels sufficient to exert biological activity, including modulation of miRNA expression in preclinical models, but also introduce risks of adverse events and drug interactions that are not observed with dietary intake alone.

Regarding adverse events, mild gastrointestinal symptoms (nausea, diarrhea, abdominal discomfort) have been reported with high‐dose quercetin and naringenin supplementation. Rare cases of hepatotoxicity have been associated with green tea extract supplements containing high concentrations of EGCG, particularly when taken on an empty stomach [180, 189, 192]. Isoflavones such as genistein are generally well‐tolerated at moderate doses [195]. Regarding drug interactions, certain flavonoids inhibit or induce cytochrome P450 (CYP) enzymes and UDP‐glucuronosyltransferases (UGTs). For example, naringenin from grapefruit juice is a well‐established inhibitor of intestinal CYP3A4, leading to increased plasma concentrations of CYP3A4 substrate drugs such as simvastatin and felodipine, thereby increasing the risk of dose‐dependent toxicity [182, 195–197]. Quercetin inhibit both CYP3A4 and UGT1A1, which may affect the metabolism of warfarin, and other narrow‐therapeutic‐index drug [182, 198, 199]. Apigenin has been shown in vitro to inhibit CYP2C9 activity and the metabolism of CYP2C9 substrates such as losartan, suggesting a potential risk of interactions with nonsteroidal anti‐inflammatory drugs [200]. Taken together, while adverse events and drug interactions are rarely reported at dietary levels of flavonoid intake, the potential for such events increases with dose and concentration, warranting particular caution in the context of high‐dose flavonoid supplementation on a case‐by‐case basis.

These restraints were addressed by proposing different novel and sophisticated delivery and formulation techniques. In this regard, to enhance metabolic stability, the structure of flavonoids can be chemically modified, offering a promising strategy to improve their bioavailability via the enhancement of solubility, metabolic stability, and membrane permeability. In pro‐drug strategies, flavonoids are chemically modified to elevate their hydrophilicity or stability; this leads to their better absorption and the subsequent conversion to the active form in target tissues. Moreover, by modifying hydroxyl groups through methylation or adding fatty acids, lipophilicity and cell membrane permeability improves, while enzymatic degradation declines, leading to the longer metabolic half‐life of flavonoids [201, 202].

An alternative proposed to circumvent the bioavailability issues of flavonoids in oral consumption is transdermal delivery. In this regard, patches or gels containing flavonoids can directly deliver these compounds into the bloodstream and bypass first‐pass hepatic metabolism. This strategy can notably enhance the flavonoid bioavailability and offer a more effective option for therapies [201]. Utilizing nanotechnology to encapsulate flavonoids in nanoparticles improves their solubility and intestinal absorption, enhances their stability, and prevents enzymatic degradation. This technique also enables targeted delivery to the tissues of interest and notably improves the bioavailability of flavonoids such as quercetin. Recent years have seen the wide usage of drug delivery systems based on nanoparticles such as solid lipid nanoparticles, liposomes, nanoemulsions, and metal nanoparticles to improve solubility and target drug delivery. To facilitate the targeted application of flavonoids, these systems can be integrated with smart and controlled drug delivery [203, 204]. To enhance the bioavailability and solubility of flavonoids, co‐solvents such as ethanol and surfactants or absorption enhancers such as piperine can be used. They exert their effect by elevating the absorption of flavonoids and preventing metabolic enzymes participating in their breakdown [201].

Although extensive in vitro research has addressed the role of polyphenols, important aspects such as metabolism and bioavailability have often been overlooked. However, concentrations of polyphenols in vivo are normally smaller than those employed in lab studies, with their molecular forms changing post absorption. Hence, *in vitro* findings based on the initial forms of polyphenols may fail to provide an accurate reflection of their actual effects in the body [205]. The emergence of technologies such as bioinformatics, omics‐based systems, molecular docking, and artificial intelligence paves the way for better understanding of interaction between flavonoids and cellular components like miRNA and optimizing their therapeutic capacity. Altogether, flavonoids are a potent group of natural compounds capable of contributing to the restoration of molecular balance in diseased states, although further research is needed. Integrating these compounds into medical practice has the potential to lead to a new class of multi‐targeted personalized medicine inspired by nature; a move away from conventional mono‐target pharmacotherapy toward safer holistic therapeutic models for the treatment of complex chronic conditions.

## 7. Conclusion

Flavonoids represent a promising class of natural compounds capable of modulating miRNA expression and exerting epigenetic regulatory effects across a wide spectrum of diseases, including cancer, diabetes, depression, and neurodegenerative disorders. Through the regulation of both tumor‐suppressive and oncogenic miRNAs, flavonoids influence key molecular pathways involved in inflammation, oxidative stress, metabolic dysfunction, and neuronal survival. Their multi‐targeted mechanisms, combined with relatively low toxicity at dietary levels, position them as attractive candidates for the development of safer and more effective therapeutic strategies. Despite these advantages, several challenges limit their clinical translation. Poor bioavailability, rapid metabolism, variability in natural sources, and the complexity of miRNA regulatory networks hinder the consistency and predictability of therapeutic outcomes. Furthermore, the majority of current evidence is derived from in vitro and animal studies, with limited validation in human clinical settings. Addressing these limitations will require well‐designed randomized clinical trials, standardized formulations, and deeper mechanistic insights into flavonoid–miRNA interactions.

Advances in nanotechnology, bioinformatics, and precision medicine offer new opportunities to overcome these barriers and optimize flavonoid‐based interventions. Future research should also explore synergistic effects with existing therapies and personalized approaches based on individual genetic and epigenetic profiles. Ultimately, integrating multidisciplinary strategies will be essential to translate flavonoid‐mediated miRNA modulation into clinically effective and reliable therapeutic applications.

**Abbreviations**
4‐HPR: N‐(4‐hydroxyphenyl) retinamide
AGO: argonaute
ALS: amyotrophic lateral sclerosis
C3G: cyanidin‐3‐glucoside
DFOG: 7‐difluoromethoxyl‐5,4 ^′^‐di‐n‐octylgenistein
DHM: dihydromyricetin
EGCG: epigallocatechin‐3‐gallate
Fla‐CN: 3‐O‐[(E)‐4‐(4‐cyanophenyl)‐2‐oxobut‐3‐en‐1‐yl]kaempferol
miRNAs: microRNAs
MQG: methoxylated quercetin glycoside
PSPD3R: purple sweet potato delphinidin‐3‐rutin
QCSPIONs: quercetin‐conjugated superparamagnetic iron oxide nanoparticles
RISC: RNA‐induced silencing complex
SFI: skullcapflavone I
TAM: tumor‐associated macrophages

## Author Contributions

Mohadese Mahdie and Sedigheh Momenzadeh participated in data collection, discussion, and writing the manuscript. Razieh Heidari and Somayeh Reiisi coordinated the study and revised the manuscript.

## Funding

This study was supported by Shahrekord University of Medical Sciences, 10.13039/501100005756, SKUMS‐8207.

## Disclosure

All authors reviewed and accepted the manuscript. Funder had no role in study design, data collection, and analysis, decision to publish, or manuscript preparation.

## Conflicts of Interest

The authors declare no conflicts of interest.

## Data Availability

The data that support the findings of this study are available from the authors upon reasonable request.
